# LMTK3 regulates breast cancer angiogenesis via a context-dependent mesenchymal-epithelial transition program

**DOI:** 10.1038/s41417-026-01001-2

**Published:** 2026-01-23

**Authors:** Jian Lu, Xiaoyan Huang, Hang Yao, Chrysa Filippopoulou, Reza Shirazi Nia, Xidong Gu, Xiaohong Xie, Qijin Shu, Georgios Giamas

**Affiliations:** 1 International Oncology Institute, The First Affiliated Hospital of Zhejiang Chinese Medical University. Oncology Department of the First Affiliated Hospital of Zhejiang Chinese Medical University, Hangzhou, China; 2 Department of General, Visceral and Transplantation Surgery, University of Heidelberg, Heidelberg, Germany; 3 Department of Breast Surgery, The First Affiliated Hospital of Zhejiang Chinese Medical University, Hangzhou, China; 4 Department of Biochemistry and Biomedicine, School of Life Sciences, University of Sussex, Brighton, UK

**Keywords:** Breast cancer, Cell biology

## Abstract

Angiogenesis constitutes a critical rate-limiting determinant of tumor progression in breast cancer (BC). Resistance to conventional anti-angiogenic therapies in BC highlights an unmet need to identify upstream molecular regulators coordinating malignant cell plasticity and vascular remodeling. Lemur tail kinase 3 (LMTK3) is a well-established oncogenic kinase; however, its specific role within the tumor angiogenic microenvironment remains undefined. Here, we identify LMTK3 as a context-dependent driver of angiogenesis through a mesenchymal-epithelial transition (MET) program. By integrating single-cell RNA sequencing with functional validation, we uncover a ‘Simpson’s paradox’ (where a correlation present in different groups disappears or reverses when combined): In mesenchymal-like triple-negative breast cancer (TNBC), LMTK3 promotes a pro-angiogenic, ‘partial EMT’ (p-EMT) state characterized by sustained ERK signaling and elevated secretion of angiogenic factors, including angiogenin. Conversely, in luminal-like cells, LMTK3 enforces a hyperepithelialized state that suppresses angiogenic phenotypes. Consequently, LMTK3 emerges as a central regulator of angiogenic plasticity, and its targeted inhibition offers a promising strategy to abrogate the pro-angiogenic p-EMT state and promote vascular normalization in TNBC.

## Introduction

Tumor-associated angiogenesis is a pivotal rate-limiting step in breast cancer (BC) progression and metastasis. Although anti-VEGF therapies demonstrate clinical utility, adaptive resistance mechanisms driven by tumor cellular plasticity represent a significant therapeutic obstacle. This underscores the imperative to identify upstream molecular targets that co-regulate malignant cell state and microenvironmental angiogenesis [1, 2]. Lemur tail kinase 3 (LMTK3) has been implicated as an oncogenic kinase in BC, regulating estrogen receptor alpha (ERα) activity and chemotherapy resistance [3–6]. However, its role in angiogenesis remains enigmatic, as high LMTK3 expression does not consistently predict clinical outcome, suggesting context(environment)-dependent functions [7, 8].

In this study, we report a novel mechanism whereby LMTK3 governs angiogenesis indirectly by functioning as an epigenetic gatekeeper-potentially through kinase-mediated signaling nodes-of mesenchymal-epithelial transition (MET) signaling in a cell lineage-specific manner. Here, we define LMTK3 as a molecular switch that promotes a pro-angiogenic partial EMT (p-EMT) state in mesenchymal-like triple-negative breast cancer (TNBC) while conversely reinforcing an anti-angiogenic hyperepithelialized state in luminal-like BC cells.

## Materials and methods

### Cell lines and culture

Human BC cell lines MDA-MB-231 (mesenchymal type, TNBC), MCF-7, and T-47D (epithelial type, Luminal A type) were cultured under standard conditions [9]. The methods for constructing LMTK3 overexpression (OE) stable cell lines and wild-type (WT) control cell lines were as previously described [9].

### Bioinformatics analysis

This study analyzed a publicly available single-cell RNA sequencing dataset (GSE176078) comprising 26 primary BC samples. Both angiogenesis and EMT scores were calculated using the AddModuleScore function in the Seurat R package, based on gene sets from the MSigDB database. Pearson correlation coefficients were employed to assess the association between LMTK3 expression and angiogenesis/EMT features, with additional analysis conducted across different clinical subtypes and EMT states.

### Angiogenesis assay

The angiogenic potential was assessed using a human umbilical vein endothelial cell (HUVEC) tubule formation assay. HUVECs were cultured for 24 hours in conditioned medium (CM) collected from either WT or LMTK3-OE BC cells. Upon collection of CM, cells were washed and cultured in serum-free medium for 24 hours. Tubular structures were imaged, and the number of nodes was quantified using ImageJ software.

### Elisa and Western blot

The levels of secreted angiogenin in CM were quantified using a human angiogenin ELISA kit. Protein expression of LMTK3, E-Cadherin, Vimentin, Zeb1, Slug, Snail, and p-ERK was analyzed using Western Blot.

### Statistical analysis

Data are expressed as mean ± standard deviation (SD). Differences were analyzed using GraphPad Prism software with Student’s *t* test or Wilcoxon test. *P* < 0.05 was considered statistically significant.

## Results

### LMTK3 expression correlates with angiogenic potential in a BC subtype-dependent manner

**Fig. 1:**
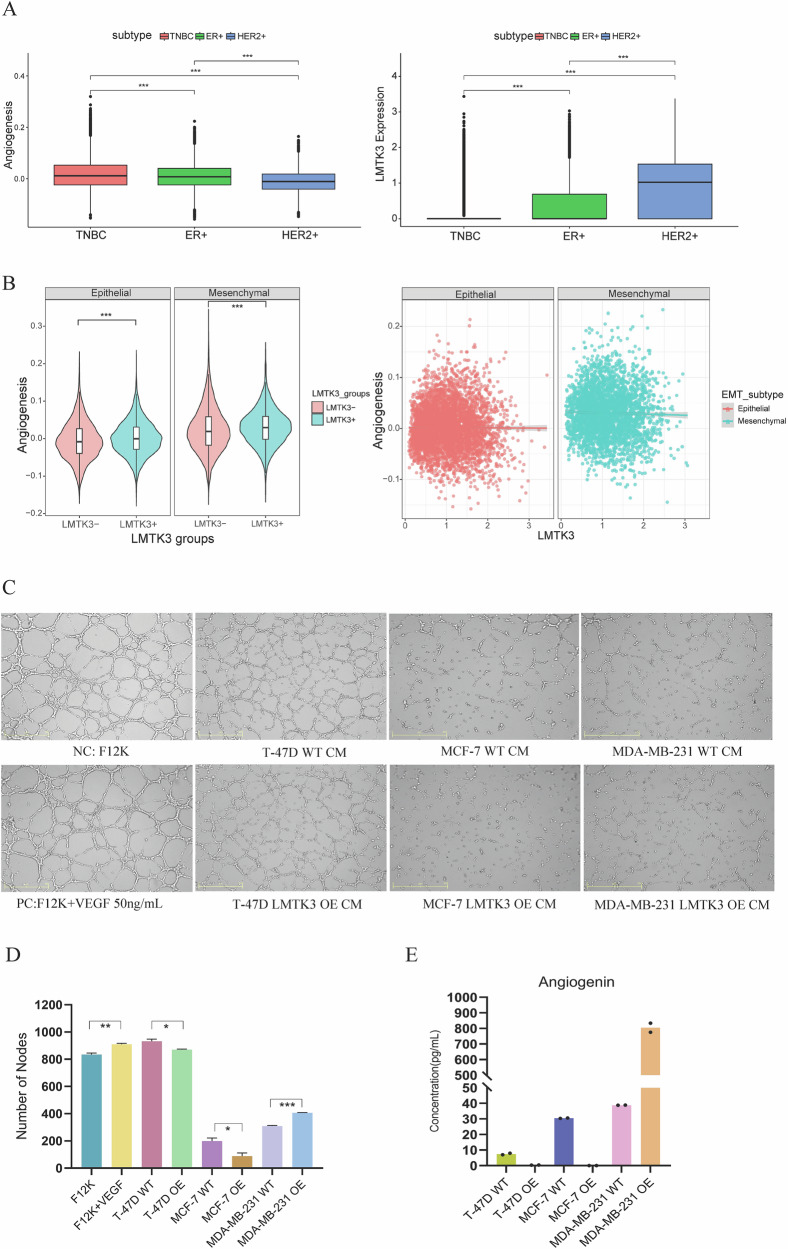
LMTK3 regulates angiogenesis in a cell-type-dependent manner. **A** scRNA-seq analysis of angiogenesis scores and LMTK3 expression across BC subtypes (TNBC, ER + , HER2 + ). (*P* < 0.001, indicated by ***). **B** Violin and scatter plots of LMTK3 expression with angiogenesis scores within each subtype (epithelial and mesenchymal). **C**, **D** HUVEC tube formation assays using CM from WT and LMTK3-OE cells. (*P < 0.05, **P < 0.01, ***P < 0.001). **E** ELISA quantification of secreted angiogenin levels in T-47D/MCF-7/MDA-MB-231 WT and LMTK3-OE cells.

Analysis of scRNA-seq data from human BC samples uncovered a ‘Simpson’s paradox’ in the relationship between LMTK3 and angiogenesis. While TNBC subtypes exhibited lower overall LMTK3 expression in a between-subtype analysis, a significant positive correlation between LMTK3 expression and angiogenesis scores was evident within the TNBC subtype itself (Fig. 1A, B).

Functional validation using HUVEC tube formation assays confirmed this context-specific nature. Compared to WT cells, CM from LMTK3-OE MDA-MB-231 cells (mesenchymal background) robustly enhanced tube formation, whereas CM from LMTK3-OE T-47D and LMTK3-OE MCF-7 cells (epithelial background) suppressed it (Fig. 1C, D). Overall, these in vitro findings validate the Simpson’s paradox, confirming that LMTK3 switches between pro-angiogenic and anti-angiogenic phenotypes depending on the cellular lineage. We hypothesized that LMTK3 remodels the tumor cell secretome to exert these paracrine effects. Indeed, ELISA assays identified angiogenin as a critical and representative mediator of this secretome shift, with its secretion being upregulated in LMTK3-OE MDA-MB-231 cells and downregulated in LMTK3-OE T-47D/MCF-7 cells (Fig. 1E), directly aligning with the angiogenic phenotypes.

### LMTK3 drives a MET program to induce a pro-angiogenic p-EMT state

**Fig. 2:**
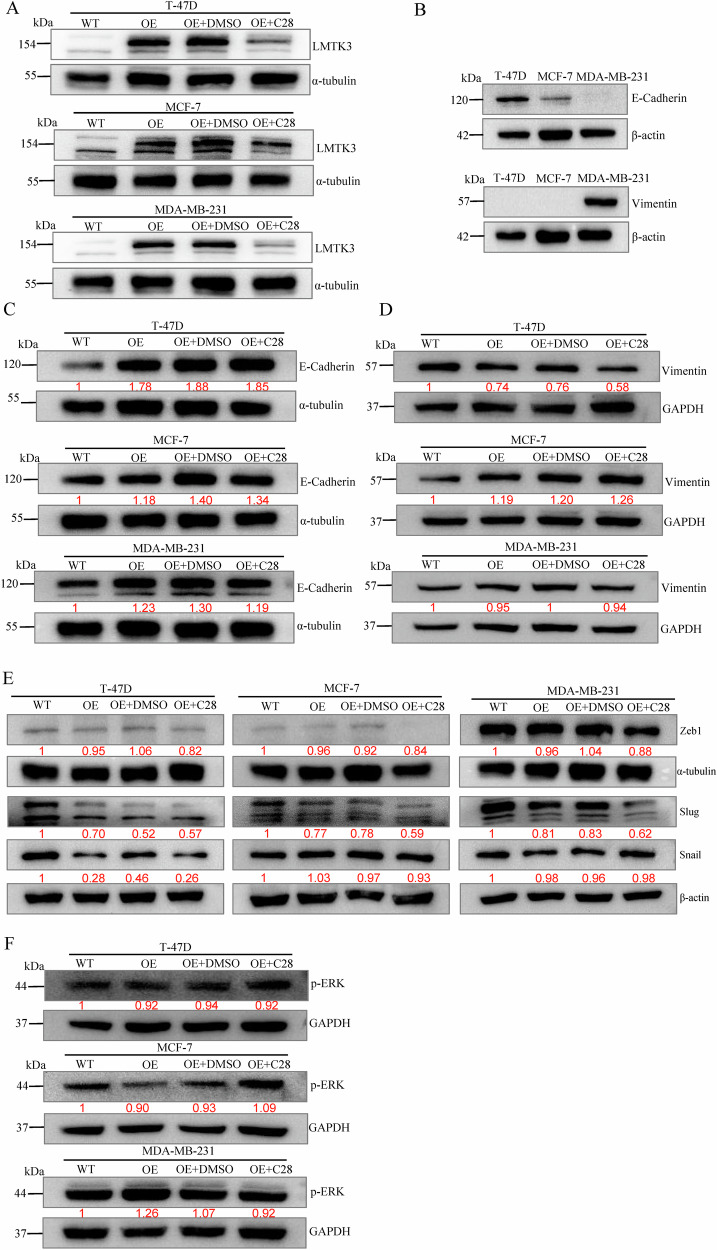
LMTK3 induces MET via the p-ERK signaling pathway and regulates the angiogenesis secretome. **A** Western blot images of LMTK3 in WT, LMTK3-OE, and C28-treated cells. **B** Western blot images of E-Cadherin and Vimentin in WT T-47D, MCF-7, and MDA-MB-231 cell lines. **C**, **D** Western blot images of E-Cadherin and Vimentin in WT, LMTK3-OE, and C28-treated cells. **E** Western blot images of core EMT transcription factors (Zeb1, Twist1, Slug, Snail) in WT, LMTK3-OE, and C28-treated BC cell lines. **F** Western blot images of p-ERK in WT, LMTK3-OE, and C28-treated BC cell lines.

We hypothesize that LMTK3 regulates angiogenesis by modulating cellular plasticity. Western blot analysis confirmed elevated LMTK3 protein levels upon LMTK3-OE, and LMTK3 suppression following treatment with the C28 inhibitor [10] (Fig. 2A). Baseline characterization confirmed T-47D and MCF-7 cells as epithelial (E-cadherin high) and MDA-MB-231 as mesenchymal (vimentin high) (Fig. 2B). Notably, LMTK3-OE promoted a conserved MET program, upregulating E-cadherin (Fig. 2C), while Vimentin exhibited different changes in different cell lines (Fig. 2D), which may suggest that the functional recovery of E-Cadherin is a key early event defining the occurrence of MET. Moreover, LMTK3-OE resulted in partial repression of certain core EMT transcription factors (Zeb1, Slug, Snail) across different BC cell lines (Fig. 2E). Interestingly, pharmacological inhibition using the C28 inhibitor in the LMTK3-OE BC cell lines did not reverse the expression levels of these proteins in most cases, suggesting potential off-target effects of C28 and/or other triggered cellular compensatory mechanisms (as discussed below).

**Fig. 3:**
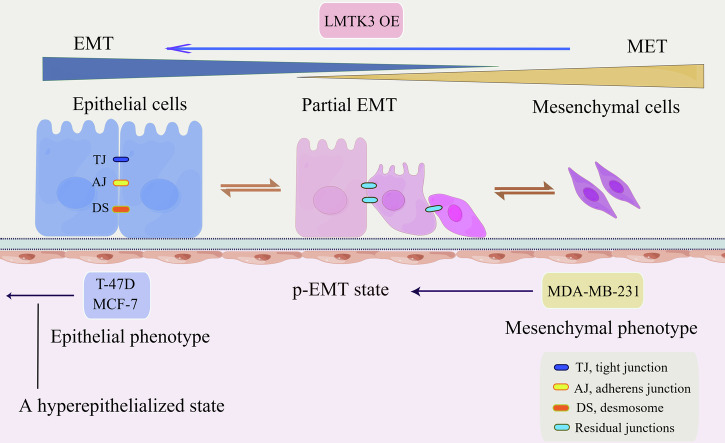
Proposed model: LMTK3 drives MET. In mesenchymal cells (M), this induces a p-EMT state. In epithelial cells (E), this induces an anti-angiogenic hyperepithelialized state [15].

However, the phenotypic outcome of this MET was lineage-dependent. In MDA-MB-231 cells, LMTK3 induced a p-EMT state characterized by co-expression of epithelial (E-Cadherin) and mesenchymal (vimentin) markers. This hybrid state was associated with peak of p-ERK signaling (Fig. 2F) and angiogenin secretion (Fig. 1E). Conversely, in luminal T-47D/MCF-7 cells, LMTK3 enforced a hyperepithelialized state (Fig. 3), resulting in suppressed p-ERK signaling (Fig. 2F) and reduced angiogenin secretion (Fig. 1E). Pharmacological inhibition using C28 attenuates the phenotype induced by LMTK3-OE, particularly in regulating p-ERK levels. However, this rescue effect exhibits context-dependent properties, suggesting that pharmacological inhibition of LMTK3 kinase activity may yield distinct signaling outcomes compared to transcriptional suppression.

## Discussion

This study resolves prior contradictions regarding LMTK3’s role in angiogenesis by identifying it as a principal upstream driver of MET in BC, providing a biological explanation for the observed “Simpson’s paradox” phenomenon. We demonstrate that LMTK3 does not directly transcriptionally regulate angiogenic genes but instead functions as a central regulator of cellular plasticity, thereby shaping the secretory profile of tumor cells in a context-dependent manner.

We propose a hierarchical model wherein the pre-existing EMT state of the tumor cell dictates the angiogenic outcome of LMTK3 activity (Fig. 3). In mesenchymal TNBC, LMTK3-driven MET induces a highly plastic, pro-angiogenic p-EMT state, aligning with evidence that such hybrid states confer heightened tumorigenic and metastatic potential compared to fully mesenchymalized or fully epithelialized cells [11–14]. Conversely, in epithelial luminal BC cells, LMTK3 reinforces a rigid hyperepithelialized state, constraining plasticity and suppressing MAPK/ERK-driven pro-angiogenic factor secretion, potentially influencing less invasive metastatic trajectories. Thus, LMTK3 operates as a contextual angiogenic switch—promoting vascularization in TNBC while suppressing it in luminal subtypes. This mechanism perfectly explains why, despite the overall low expression of LMTK3 in TNBC, its expression level still correlates positively with angiogenesis scores.

Notably, despite LMTK3-OE persistently driving the MET program, functional reversal by the C28 inhibitor exhibits complexity across different cell lines. Unlike gene knockdown, C28 is an ATP-competitive inhibitor. Given the multi-target pharmacology typical of kinase inhibitors, C28 may exert off-target effects on other kinases regulating the EMT/MET spectrum. This may explain why C28 treatment did not reverse the LMTK3-OE phenotype across all molecular markers. Specifically, the incomplete reversal of E-cadherin or p-ERK suggests that LMTK3 may also function through scaffold protein interactions independent of its catalytic activity, or that C28-mediated inhibition triggered compensatory signaling pathways within the angiogenic microenvironment. Ultimately, in order to delineate the mechanistic involvement of LMTK3 in MET, subsequent investigations should employ genetic perturbation via siRNA-mediated knockdown and/or CRISPR-Cas9 knockout.

From a translational perspective, LMTK3 inhibition represents a promising therapeutic strategy in TNBC that extends beyond direct cytotoxicity. However, this context-dependent nature presents a therapeutic paradox for luminal BC. While LMTK3 inhibition benefits targeting the ERα signaling pathway and cell proliferation, our data suggest that such inhibition could theoretically inadvertently release the “super-epithelial” inhibition of angiogenesis, thereby promoting vascular remodeling. This suggests that in luminal subtypes, optimal efficacy may require combination therapy with anti-angiogenic agents (e.g., bevacizumab) to counteract this potential side effect. By blocking the MET driver, such inhibition could force tumor cells to exit the pro-angiogenic p-EMT state, thereby suppressing neovascularization or promoting tumor vascular normalization.

Future research should validate this “vascular normalization” hypothesis in TNBC-derived patient-derived xenograft models. Specifically, the efficacy of combining the LMTK3 inhibitors with standard chemotherapy (e.g., paclitaxel) should be evaluated to determine whether LMTK3 inhibition can improve tumor perfusion, alleviate hypoxia, and enhance chemotherapeutic delivery.
